# Insights into *CYP1B1*-Related Ocular Diseases Through Genetics and Animal Studies

**DOI:** 10.3390/life15030395

**Published:** 2025-03-03

**Authors:** Elizabeth M. Bolton, Andy Drackley, Antionette L. Williams, Brenda L. Bohnsack

**Affiliations:** 1 Division of Ophthalmology, Ann & Robert H. Lurie Children’s Hospital of Chicago, 225 E. Chicago Ave, Chicago, IL 60611, USA; ebolton@luriechildrens.org (E.M.B.); anwilliams@luriechildrens.org (A.L.W.); 2 Department of Ophthalmology, Northwestern University Feinberg School of Medicine, 645 N. Michigan Ave, Chicago, IL 60611, USA; 3 Department of Pathology and Laboratory Medicine, Ann & Robert H. Lurie Children’s Hospital of Chicago, 225 E. Chicago Ave, Chicago, IL 60611, USA; adrackley@luriechildrens.org

**Keywords:** *CYP1B1*, primary congenital glaucoma, anterior segment dysgenesis, Peters Anomaly, Axenfeld–Rieger Syndrome, juvenile open-angle glaucoma, primary open-angle glaucoma

## Abstract

The *CYP1B1* gene encodes a cytochrome p450 monooxygenase enzyme, and over 150 variants have been associated with a spectrum of eye diseases, including primary congenital glaucoma, anterior segment dysgenesis, juvenile open-angle glaucoma, and primary open-angle glaucoma. Clinical genetics has yielded insights into the functions of the various *CYP1B1* gene domains; however, animal studies are required to investigate the molecular role of *CYP1B1* in the eye. While both zebrafish and mice express *CYP1B1* in the developing eye, embryonic studies have shown disparate species-specific functions. In zebrafish, *CYP1B1* regulates ocular fissure closure such that overexpression causes a remarkable phenotype consisting of the absence of the posterior eye wall. Adult *CYP1B1* null zebrafish lack an ocular phenotype but show mild craniofacial abnormalities. In contrast, *CYP1B1*^−/−^ mice display post-natal mild to severe trabecular meshwork degeneration due to increased oxidative stress damage. Interestingly, the retinal ganglion cells in *CYP1B1* null mice may be more susceptible to damage secondary to increased intraocular pressure. Future studies, including detailed genotype–phenotype information and animal work elucidating the regulation, substrates, and downstream effects of *CYP1B1*, will yield important insights for developing molecularly targeted therapies that will aim to prevent vision loss in *CYP1B1*-related eye diseases.

## 1. Introduction

**Figure 1:**
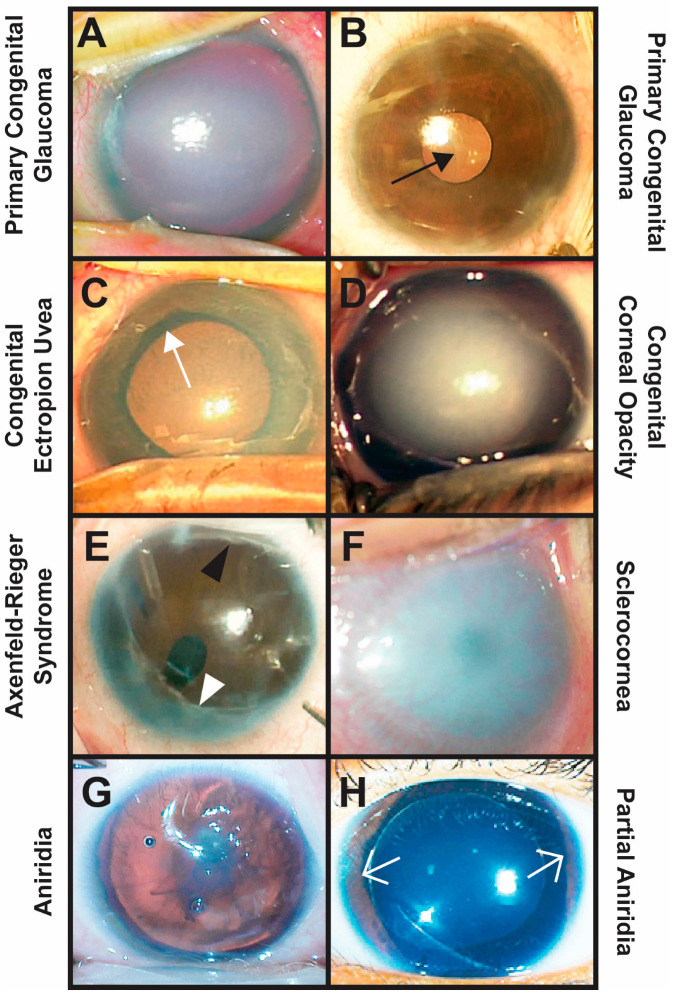
Clinical phenotypes of *CYP1B1*-related congenital eye diseases. (**A**,**B**) Primary congenital glaucoma is diagnosed between birth and 3 years of age and is characterized by elevated IOP typically due to trabeculodysgenesis. Importantly, there is an absence of other types of anterior segment dysgenesis. Typical clinical signs include buphthalmos with increased corneal diameter and axial length, corneal edema (**A**), Haabs striae ((**B**) black arrow, breaks in Descemets membrane), and glaucomatous optic neuropathy. (**C**) Congenital ectropion uvea occurs due to the failure of remnant neural crest cells within the anterior segment to undergo apoptosis. This results in a membrane that pulls the pigmented iris epithelium through the pupil (ectropion uvea, white arrow) and covers the iris and angle. Glaucoma is common and may initially be due to trabeculodysgenesis, but eventually, the membrane causes angle closure. (**D**) Congenital corneal opacities are considered Peters Anomaly if there is an absence of Descemet’s membrane underlying the corneal defect. Peters Anomaly is due to abnormal separation of the lens vesicle from the overlying surface ectoderm and is divided into two types based on whether the lens is involved (Type 2) or not (Type 1). Other congenital corneal opacities not classified as Peters Anomaly are typically avascular, and the Descemet’s membrane is present under the corneal stromal haze. Glaucoma is diagnosed in more than 50% of affected individuals and occurs due to trabeculo-iridogoniodysgenesis and/or angle closure. (**E**) Axenfeld–Rieger Syndrome is characterized by Axenfeld Anomaly (posterior embryotoxon (black arrowhead) with iridocorneal touch (white arrowhead)) and Rieger anomaly (iris hypoplasia with pseudopolycoria and/or corectopia). More than 50% of affected individuals develop glaucoma due to iridogoniodysgenesis. (**F**) Sclerocornea is the absence of demarcation between the cornea and sclera resulting in diffuse corneal opacification often with neovascularization. Sclerocornea is often accompanied by congenital aphakia and glaucoma due to trabeculo-iridogoniodysgenesis. (**G**,**H**) Aniridia classically shows pan-ocular defects, including foveal hypoplasia, optic nerve dysplasia/hypoplasia, iris hypoplasia, cataract, and keratopathy, due to limbal stem cell deficiency. In partial aniridia, there is remnant iris (white open arrows) with varying degrees of other ocular findings. Both types are associated with open-angle glaucoma (iridogoniodysgenesis) or closed-angle glaucoma (anterior rotation of the remnant iris root).

Variants in the *CYP1B1* gene are most commonly associated with primary congenital glaucoma (PCG) but have also been identified in other congenital eye diseases, such as congenital ectropion uvea (CEU), congenital corneal opacification (CCO), Axenfeld–Rieger Syndrome (ARS), sclerocornea, and aniridia (Figure 1) [1,2,3,4,5,6]. These diseases are collectively referred to as anterior segment dysgeneses, and in most, there is a disruption in the migration or differentiation of ocular neural crest cells, a transient embryonic stem cell population that contributes to the corneal stroma and endothelium, sclera, iris stroma, ciliary body stroma, and trabecular meshwork [5,7,8]. In addition, *CYP1B1* variants have been found in juvenile open-angle glaucoma (JOAG) and adult-onset primary open-angle glaucoma (POAG) [2,3,9,10]. These potentially blinding diseases are clinically distinct, but their common link with *CYP1B1* variants demonstrates the importance of this gene in the development and maintenance of ocular anterior segment structures.

As such, herein, we review the association of *CYP1B1* variants in congenital and childhood eye diseases and our current understanding of its molecular mechanisms from zebrafish and mouse studies. A comprehensive and systematic PubMed literature review was conducted that captured the current state of clinical knowledge and research on the *CYP1B1* gene. We then synthesize this information to propose how this work can potentially contribute to molecularly targeted treatments that will prevent vision loss in affected children.

## 2. *CYP1B1* Gene

**Figure 2:**
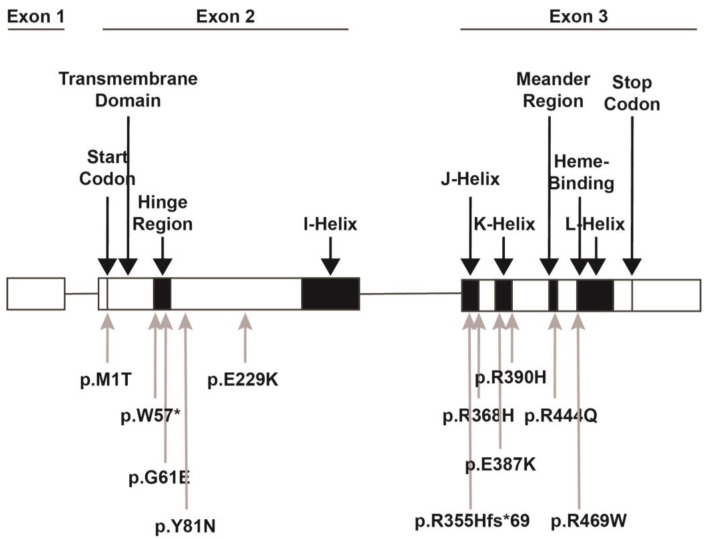
*CYP1B1* gene structure. The *CYP1B1* gene consists of 3 exons with the start codon in the 2nd exon followed by the N-terminal transmembrane domain. The hinge region gives flexibility to the remaining cytosolic globular portion of the protein. The conserved core structures include the four helices (I-, J-, K-, and L-), between which the meander region and the heme binding sites are located. The location of variants discussed within this review are denoted. The p.M1T variant disrupts the start codon, thereby preventing translation. The p.W57* and G61E variants are both within the hinge region. The W57* is a nonsense variant, while the G61E variant leads to decreased enzymatic activity. The p.Y81N and p.E229K variants are between the hinge region and the I-helix and are predicted to decrease enzymatic activity and disrupt the helical structure, respectively. The p.R355Hfs*69, p.R368H, p.E387K, and p.R390H variants are within the J- and K-helices. The p.E387K and p.R390H variants decrease protein stability and enzymatic activity. The p.368H variant is a VUS but is predicted to have decreased enzymatic activity. The p.R444Q variant in the meander region and the p.R469W variant in the heme-binding region both decrease heme binding, thereby inhibiting protein activity.

**Figure 3:**
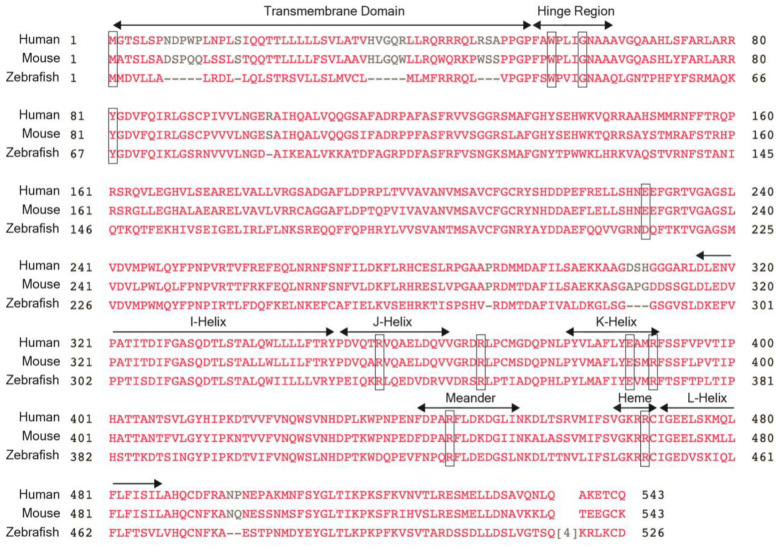
*CYP1B1* protein sequence. The protein sequences for human *CYP1B1* show 81% and 56% homology with mouse and zebrafish *CYP1B1*, respectively. There is a high level of conservation between the 3 species in the hinge region, the four helices (I-, J-, K- and L-), the meander region, and the heme-binding domain, which are all denoted. Thus, clinically relevant variants tend to be clustered within these regions. Notably, the amino acids affected by clinically relevant variants discussed within this review, namely W57, G61, R355, R368, E387, R390, R444, and R469 (denoted by boxes), are also conserved between humans, mice, and zebrafish.

The *CYP1B1* gene is located on 2p22-p21 and consists of three exons and two introns, with the open reading frame starting in the second exon (Figure 2) [11,12,13]. The encoded *CYP1B1* protein is a cytochrome p450 heme-thiolate monooxygenase enzyme that oxidizes both endogenous and exogenous substrates [14,15]. The protein, which is 543 amino acids long, is localized to the endoplasmic reticulum and is composed of a membrane-bound N-terminal region which is bridged with the cytosolic globular domain by a hinge region (Figure 3). The cytosolic globular domain contains the substrate-binding region and the conserved core structures (CCSs) that include the I-, J-, K-, and L-helices, with the meander region lying between the K- and L-helices and the heme-binding site just 5’ to the L-helix [12,14,16,17]. Through the CCS region, the protein binds a complement reductase enzyme and a heme molecule which are required for oxidizing the targeted substrate [16,17,18]. Identified endogenous enzymatic reactions mediated by *CYP1B1* include the conversion of retinol to retinoic acid (RA), 17ß-estradiol to 2-hydroxy and 4-hydroxy estradiol, testosterone to 6ß-hydroxytestosterone, melatonin to 5-hydroxy melatonin, and arachidonic acid to mid-chain and terminal hydroxyeicosatetraenoic (HETEs) and epoxyeicosatrienoic (EETs) acids [19,20,21,22,23,24,25]. Thus, *CYP1B1* has diverse roles in regulating the synthesis of signaling molecules (e.g., retinoic acid, HETEs, and EETs) and the metabolism of hormones (e.g., estradiol, testosterone, and melatonin). Furthermore, *CYP1B1* metabolizes exogenous procarcinogen toxins, namely polycyclic aromatic hydrocarbons [26,27]. As such, the majority of published *CYP1B1* studies have focused on its function in various types of cancer such as breast, ovarian, prostate, lung, and colon [28]. Nevertheless, the molecular role of *CYP1B1*, including targeted substrates, in the embryonic and adult eye have yet to be fully elucidated.

## 3. *CYP1B1* Genetics in Human Eye Diseases

The *CYP1B1* gene was initially designated as the GLC3A locus by candidate regional and general positional mapping of 11 families with PCG [11]. The locus was then identified as the *CYP1B1* gene in 1997 [13]. Subsequently, over 150 disease-causing variants comprising predominantly missense, nonsense, and frameshift have been identified [2,3,6,17]. In this setting, anterior segment dysgenesis (e.g., PCG, CEU, CCO, ARS, sclerocornea, and aniridia) and JOAG are typically autosomal recessive and associated with homozygous or compound heterozygous missense or truncating variants. In contrast, heterozygous missense variants have been identified in adults with POAG [2,3,9,10].

Due to founder effects within certain populations, there are a few common missense variants which account for a significant proportion of PCG cases [29]. For example, in the Slovakian Roma population, PCG is 16 times more frequent (1:1250) than in Western Europe and the United States (1:20,000), and the majority of cases are due to a lysine for glutamic acid substitution at the 387 location (p.E387K) [30,31,32,33]. Similarly, the variant p.R390H is commonly found within Middle Eastern (Saudi Arabian and Iranian), Indian, and Chinese populations [34,35,36]. The glutamic acid at position 387 forms a crucial salt bridge with arginine at position 390 within the K-helix such that an alteration in either of these sites decreases protein stability (especially within the meander region) and lowers enzymatic activities [16,35,37]. Genotype–phenotype studies have shown that these variants cause a severe PCG phenotype that is refractory to angle surgery (trabeculotomy and goniotomy) [36]. Furthermore, the p.R390H variant in particular has been associated with bilateral CEU, severe neonatal-onset glaucoma, and corneal decompensation in an Indian population [38]. The p.R469W was also found within Saudi Arabian, Iranian, and Turkish populations and is located within the heme-binding region [19,39,40,41,42]. The exact mechanism underlying the pathogenic nature of the p.R469W variant is unknown; however, the phenotype in general is severe PCG with poor prognosis (multiple surgeries, corneal decompensation or scarring, and phthisis bulbi) [19,40,43]. Interestingly, R469 is only conserved amongst the CYP1 family and is adjacent to the invariable cytosine at position 470, suggesting that a basic and positively charged amino acid is required in that location for heme binding. In contrast, the glycine to glutamic acid substitution at residue 61 (p.G61E), which is common in the Middle East, Morocco, Turkey, and Brazil, is within the hinge region and is located at the opening of the substrate access channel. This variant is suspected to inhibit substrate binding and subsequent enzymatic activity, although less overall protein has also been detected, suggesting either transcript or protein instability [1,19,44,45,46,47]. The resultant PCG phenotype is reportedly more mild, typically responding to angle surgery. The G61, E387, and R390 amino acids are highly conserved amongst cytochrome p450 family members, further confirming their essential role in protein function.

While anterior segment dysgenesis is more commonly associated with autosomal dominant inheritance and heterozygous variants in the *FOXC1*, *PITX2*, and *PAX6* genes, pathogenic *CYP1B1* variants have also been reported in these more severe congenital eye diseases [5,6,8,48,49]. Notably, there is variable expressivity noted with *CYP1B1* variants, with family members often exhibiting different phenotypes. Nevertheless, there are a few notable trends in these patients with anterior segment dysgenesis, namely a high frequency of variants affecting the 5’ end of the gene and variants resulting in premature truncation. For example, the prevalent c.171G > A variant results in the substitution of W57 located in the hinge region with a stop codon (p.W57*). This variant, often in combination with a second nonsense or frameshift variant in trans, has been identified in patients with CCO and ARS [50,51]. Another variant, p.R355Hfs*69, is seen due to a 13-base pair deletion from nucleotide 1064 to 1076 (c.1064_1076del), and has been identified in patients with PCG, JOAG, CCO, and ARS [3,51,52,53]. The subsequent frameshift leads to a premature stop codon that results in a non-functional truncated protein missing the K-helix, meander, heme-binding, and L-helix regions and/or the activation of nonsense-mediated mRNA decay. The G61E variant, especially when found in the homozygous state, has also been identified in patients with CCO, ARS, and sclerocornea [3,54]. The PCG phenotype associated with G61E is relatively mild compared to the p.E387K and p.R390H variants, yet it has been reported in these more severe anterior segment dysgenesis phenotypes. The basis for this phenotypic variation remains unknown but may be due to the influence of polymorphisms in other genes associated with childhood glaucomas, such as *PITX2*, *FOXC1*, *PAX6*, and *MYOC*; the epigenetic regulation of the *CYP1B1* gene; or post-translational modifications on protein synthesis and function [5,55,56].

In addition to these congenital eye diseases, compound heterozygous *CYP1B1* variants have also been reported in patients with JOAG, which is defined as open-angle glaucoma without anterior segment dysgenesis diagnosed between 3 and 40 years of age [9,37,53]. In some families, siblings with the same pathogenic variants display disparate phenotypes such as congenital corneal opacity in one individual and JOAG in another [44]. One interesting variant, R368H, which has been identified in PCG, anterior segment dysgenesis, JOAG, and POAG, is classified as a variant of uncertain significance (VUS) due to conflicting findings ranging from benign to pathogenic [50,52,53,57,58]. In our practice, two unrelated patients diagnosed with JOAG were found to have compound heterozygous *CYP1B1* variants, with one allele being the p.R368H variant. One patient, who was diagnosed with JOAG at 5 years old and required bilateral Baerveldt glaucoma drainage devices to obtain IOP control, had both the p.R355Hfs*69 and p.R368H variants. The other patient who was diagnosed with JOAG at 8 years old had the p.W57* and p.R368H variants. IOP control in this patient was achieved with angle surgery. The arginine at position 368 is located between the J- and K-helices. In vitro evidence suggests that the arginine to histidine substitution results in decreased protein expression and subsequent impaired oxidation of retinol to retinoic acid and metabolism of 17ß-estradiol [43]. On the other hand, there are reports of non-symptomatic individuals who are homozygous for the p.R368H variant, and gnomAD shows that this variant is relatively common with a frequency of 2–3% in South Asian, Middle Eastern, and Ashkenazi Jewish populations [59,60]. Thus, there are likely many individuals who are homozygous for this variant that are unaffected, mildly affected, or potentially pre-symptomatic. As a result, the p.R368H variant is hypothesized to have a mild effect with low penetrance, but when combined with a second pathogenic variant in trans, especially one that results in premature truncation or loss of protein expression (as seen in the two patients in our study), it can result in disease.

Adult-onset POAG is multi-factorial, and genome-wide association studies (GWAS) have identified numerous genes with various levels of pathogenicity. Many *CYP1B1* missense, nonsense, and frameshift variants associated with PCG, anterior segment dysgenesis, and JOAG have also been reported as heterozygous in patients with POAG [2,9,10,17,61,62]. One variant in which tyrosine is replaced by asparagine at the 81st amino acid location (p.Y81N) has been identified in a handful of patients with POAG, as well as in individuals with PCG [9,10]. This tyrosine is a conserved amino acid, and the substitution decreases enzymatic activity. However, this variant is also classified as a VUS due to reports of non-symptomatic individuals who carry this variant. Like the p.R368H variant, this hypomorphic allele may have low penetrance, or individuals may be pre-symptomatic in the context of POAG.

The identification of *CYP1B1* variants in patients with PCG, anterior segment dysgenesis, JOAG, and POAG gives information about the important regions for gene function and the necessity of the protein for the development of the anterior segment of the eye and the maintenance of the aqueous outflow pathways. Additional human studies are limited due to the accessibility of tissues, although *CYP1B1* mRNA and *CYP1B1* protein have been detected in numerous fetal and adult human ocular tissues [63]. Interestingly, the *CYP1B1* protein was not detected by immunohistochemistry in either the fetal or adult trabecular meshwork endothelial cells but was found in the adjacent ciliary body stroma in the fetal eye and in the non-pigmented ciliary epithelium, iris pigmented epithelium, and retina in both fetal and adult eyes [63]. Based on expression data, *CYP1B1* is hypothesized to regulate the production of paracrine signals by the non-pigmented ciliary epithelium, which are then secreted into the aqueous humor and target the trabecular meshwork. Studies have shown that the *MYOC* gene, which is associated with autosomal dominantly inherited JOAG, is a downstream target of *CYP1B1* within trabecular meshwork cells. However, the *CYP1B1* substrate that mediates this interaction is unknown [56]. Thus, in order to gain more insights into *CYP1B1*-related eye diseases and better understand the molecular function of this gene, the use of animal models is required. Importantly, there is a conservation of the protein sequence between humans, mice, and zebrafish, especially within critical domains including the hinge region, four helices, meander region, and heme-binding domain (Figure 3).

## 4. *CYP1B1* in Zebrafish Eye Development

The advantage of using zebrafish is the ability to visualize developmental processes in real time as the embryos are external to the mother’s body [64]. Overall, the zebrafish eye shares genetic conservation with the mammalian eye, but there are a few anatomical differences. Given its aquatic habitat, the lens is closely apposed to the cornea in order to act as one refracting unit. Furthermore, the zebrafish eye does not have a trabecular meshwork, but rather a ventral canalicular network which then connects to the aqueous plexus and choroidal veins [65,66].

The majority of studies involving *CYP1B1* in zebrafish focus on its function in metabolizing environmental toxins [67]. Few studies to date have specifically investigated the role of *CYP1B1* in the developing eye [68,69,70]. In zebrafish embryos, *CYP1B1* is expressed in a dorsal–ventral pattern within the proximal primitive retina around the inferior and superior ocular fissures as well as in the pharyngeal arches by 24 h post fertilization (hpf). Expression within the eye decreases with the closure of the ocular fissures and is absent by 72 hpf. Further *CYP1B1* expression within the developing eye and brachial arches is inversely regulated by RA such that exogenous treatment with a retinaldehyde dehydrogenase inhibitor (N,N-diethylaminobenzaldehyde (DEAB)) increases *CYP1B1* expression [68]. Morpholino oligonucleotide knockdown of *CYP1B1* protein translation caused premature closure of the ocular fissure, which is hypothesized to hamper the later migratory neural crest cells that traverse the ocular fissure into the anterior segment. However, two different *CYP1B1* zebrafish knockouts (p.C317Sfs*23 and p.H179Gfs*6) showed no ocular defects as adults [69,70]. This may be due to the developmental and structural differences in aqueous outflow structures between zebrafish (dorsal and ventral canalicular network) and humans (trabecular meshwork). While neural crest cells migrate into the human eye in three distinct waves, with the ones forming the trabecular meshwork in the last group, in the zebrafish eye, neural crest cells migrate as a continuous stream. In addition, neural crest cells migrate in two pathways, through the ocular fissure and between the surface epithelium and distal edge of the optic cup directly into the developing anterior segment [71]. Furthermore, fate mapping studies conducted in our lab have shown that while *foxd3*-positive neural crest cells contribute to the corneal stroma and endothelium and the iris, they are sparse within aqueous outflow canalicular networks (unpublished data). Nevertheless, the p.H179Gfs*6 null zebrafish as adults showed mild craniofacial abnormalities affecting the quadrate and palatoquadrate bones within the jaw, which may be due to the lack of the early expression of *CYP1B1* in the pharyngeal arches [70]. A corresponding human phenotype has not been reported in patients with *CYP1B1* variants, although this mild jaw abnormality may be subclinical and therefore go undetected.

On the other hand, the overexpression of *CYP1B1* through mRNA injection at the one-cell stage resulted in prominent colobomas/posterior wall defects with loss of the sclera and retinal pigment epithelium. However, the retina in the area of the wall defect differentiated into a well-defined outer nuclear layer (photoreceptors), outer plexiform layer, inner nuclear layer, inner plexiform layer, and ganglion cell layer. Furthermore, the overexpression of *CYP1B1* delayed laminin breakdown within the ocular fissure and disrupted *foxd3*-positive neural crest cell migration through the ocular fissure [68]. *CYP1B1* has been shown to mediate the production of RA in an retinaldehyde dehydrogenase-independent pathway; however, the effect of *CYP1B1* overexpression was not rescued or improved by pharmacologically inhibiting RA synthesis with DEAB [21,68]. This suggests that the *CYP1B1* action within the developing eye is independent of RA; however, the specific substrate has yet to be identified. Importantly, the overexpression of zebrafish *CYP1B1* mRNA containing mutations corresponding to clinically reported variants (p.M1T, p.E229K, and p.R444Q) did not show any abnormal phenotype [68]. The p.M1T variant has been identified in association with Peters Anomaly and mutates the translation initiation codon, thereby preventing or at least disrupting the translation of the protein [72]. The p.E229K variant, which has been identified in multiple patients with phenotypes spanning the *CYP1B1* spectrum of disease, is between the hinge region and the I-helix and disrupts the helix structures [18,33,58]. However, like the p.R368H variant, the p.E229K variant has been classified as a VUS as it has a relatively high allele frequency in the general population and has also been identified in unaffected patients [3]. The p.R444Q variant affects the meander region between the K- and L-helices altering heme binding and has been identified in patients with PCG and ARS [24,73]. Thus, there is likely functional homology between zebrafish *CYP1B1* and human *CYP1B1*, although the overexpression or over-activation of the enzyme has not been seen clinically. Although zebrafish yield the ability to visualize in vivo development in real time, the differences in anterior segment anatomy, especially the lack of a trabecular meshwork, dictate the need for further studying *CYP1B1* function in mice.

## 5. *CYP1B1* in Mouse Eye Development

The mouse anterior segment is anatomically similar to the human eye, having a trabecular meshwork within the iridocorneal angle that drains the aqueous humor. In mice embryos, *CYP1B1* is expressed within the optic cup in a dorsal–ventral pattern and in the pharyngeal arches; however, unlike zebrafish, the transcripts are localized to the distal edge of the optic cup that interacts with the overlying surface ectoderm [68,74,75,76]. This hinge region of the optic cup will give rise to the ciliary body and iris, and like in humans, *CYP1B1* is expressed within the non-pigmented epithelial layer both in the embryo and in the adult mouse, but not within the trabecular meshwork [63]. Later during development and in adulthood, *Cyb1b* is expressed within the neural retina.

The first report of *CYP1B1*^−/−^ mice showed no differences in intraocular pressure over the first year of life compared to wildtype littermates. However, a histological analysis demonstrated focal angle abnormalities including defects in Schlemm’s canal, the membrane over the trabecular meshwork, and anterior insertion of the iris. Notably, crossing the *CYP1B1*^−/−^ mice into an albino (*Tyr*^−/−^) background worsened the angle dysgenesis, which was partially rescued by exogenous L-dopa administration [77]. The interaction between these two pathways is not well understood but may be due to L-Dopa reducing oxidative stress. Additional studies have demonstrated that *CYP1B1*^−/−^ mice on a different background initially had normal trabecular meshwork at birth but exhibited degeneration with elevated intraocular pressure by 3 weeks of age [78]. By 8 months of age, the trabecular meshwork had collapsed and was atrophic in these *CYP1B1*^−/−^ mice.

Interestingly, trabecular meshwork cells isolated from the *CYP1B1*^−/−^ mice showed increased oxidative stress that was mitigated by exogenous antioxidants. Similarly, in vivo, the external delivery of N-acetylcysteine, an antioxidant, during the first 3 weeks of life prevented trabecular beam atrophy, decreased apoptosis, and maintained the trabecular meshwork architecture [78,79]. The *Postn* gene, which encodes an extracellular matrix protein required for collagen fibril assembly and maturation, is an indirect downstream target of *CYP1B1* in the trabecular meshwork [78]. Further studies showed that a pericyte-specific (*Pdgfrb*) conditional knockout, but not an endothelial-specific (*VE-Cad*) knockout, also showed similar trabecular meshwork abnormalities, suggesting that the direct effect of *CYP1B1* is localized to pericytes [80]. Thus, *CYP1B1* decreases oxidative stress within the trabecular meshwork, albeit the direct substrates of this cytochrome p450 enzyme within the eye have yet to be identified. One possibility is that *CYP1B1* may regulate the production of EETs from arachidonic acid, which have been shown to decrease oxidative stress in endothelial cells and neuronal tissues [81,82]. Nonetheless, this mechanism may account for PCG that develops after the neonatal period, such as JOAG and POAG, but it does not account for the pathogenesis of anterior segment dysgenesis, which is also associated with *CYP1B1* variants. Although phenotypes such as ARS and Peters Anomaly are more rarely associated with *CYP1B1* variants, they demonstrate a role for this gene in development and not only the maintenance of the postnatal trabecular meshwork.

While abnormalities in the trabecular meshwork are central in regulating intraocular pressure, in glaucoma, irreversible vision loss is typically a result of retinal ganglion cell loss. As *CYP1B1* is expressed within the embryonic and post-natal retina, additional mouse studies have investigated the enzyme’s effect within this tissue [80,83,84]. Retinal astrocytes obtained from *CYP1B1*^−/−^ mice were more proliferative and migratory in vitro and showed an altered expression of genes important in regulating axon guidance; however, *CYP1B1*^−/−^ mice developed normal retinal ganglion cell projections through the optic nerve to the superior colliculus [83,84]. Remarkably, in response to elevated intraocular pressure, *CYP1B1*^−/−^ mice displayed worse degradation of axonal transport and the degeneration of retinal ganglion cells than their wildtype counterparts [85]. This suggests that in addition to the disruption of aqueous outflow leading to increased intraocular pressure, *CYP1B1* also plays a role in preserving retinal ganglion cell integrity in the context of ocular hypertension.

Notably, there is a difference in phenotypes between zebrafish and mice. This is likely due to the anatomic differences in aqueous humor outflow with a ventral canalicular network in fish and trabecular meshwork in mice. Also, while the gene is expressed in the developing eye, the localization within the optic cup (around the optic fissure in zebrafish and at the hinge region in mice) differs between the two species. Although the mouse phenotype is more similar to the human disease, the zebrafish model is still helpful for studying the molecular function and substrates of the *CYP1B1* enzyme within ocular tissues.

## 6. Future Directions

Molecularly targeted therapies have become reality within ophthalmology with the advent of gene therapies for retinal dystrophies [86]. Gene therapy has progressed significantly over the past 15 years with approaches that include gene replacement and in situ gene silencing and editing [87]. The retina, in particular, was an attractive site for initial studies due to is immune-privileged status. Nevertheless, in some patients treated with voretigene neparvovec-rzyl, the only currently FDA-approved retinal gene therapy, the adenovirus-based vector has caused localized immune responses [88]. This has triggered the development of non-viral-based vectors such as liposomes and nanoparticles, especially as the targeting of anterior segment diseases has become of greater interest [87,89,90]. In particular, lipid nanoparticles have been shown to target corneal endothelium and the trabecular meshwork in mice [90]. Furthermore, unlike adult open-angle glaucoma, which is often multi-genic and multi-factorial, pediatric glaucomas are often mono-genic, making them an excellent gene therapy target. Thus, *CYP1B1* could be an attractive gene therapy target in the anterior segment whether it be gene replacement or editing.

However, a challenge with *CYP1B1*-related eye diseases is the phenotypic variability and the typically autosomal recessive mode of inheritance such that genetic diagnosis is often lacking or made long after disease onset and progression [17]. In addition, there is a need for more in-depth genotype–phenotype information, specifically the response to glaucoma surgeries for the more than 150 *CYP1B1* variants. The first step is to increase genetic testing, which can be especially difficult in the United States due to the denial of insurance coverage. The subsequent dissemination of genotype–phenotype data regarding *CYP1B1*-related eye diseases can help guide pediatric ophthalmologists and glaucoma specialists to best practices, especially regarding the choice of IOP-lowering surgery. Additional animal studies and in vitro human studies using cultured primary or stem cell-derived trabecular meshwork cells are necessary for identifying specific *CYP1B1* substrates and downstream targets.

Other non-gene therapies, such as molecularly targeted treatments, could also be developed for *CYP1B1*-related eye diseases. The most attainable goal at this time is mitigating oxidative stress. While the beneficial effects of antioxidants on the trabecular meshwork in childhood glaucomas have yet to be described, various antioxidants are currently undergoing clinical trials for adult-onset glaucomas, and in vitro studies have shown the positive effects of antioxidants on cultured trabecular meshwork cells [91,92]. Thus, once there are better data as to *CYP1B1* substrates within the trabecular meshwork, it is reasonable to consider targeted antioxidants to mitigate the effect of a defective enzyme. Nonetheless, as more human data are obtained regarding *CYP1B1*-related eye diseases, animal studies will continue to be at the forefront for understanding the multi-faceted disease pathogenesis and are necessary for eventually developing new meaningful treatments.

As a panel of authors, we represent experienced surgeons in the field of pediatric glaucomas, experts in gene variant interpretation, as well as developmental biologists who study zebrafish anterior segment formation. Thus, together, we offer a unique vantage point and insights that combine the clinical and basic science of *CYP1B1*-related pediatric ophthalmologic diseases, and we are uniquely poised to understand the significance of the genotype–phenotype associations and molecular animal studies focused on this gene. Nevertheless, bias regarding the inclusion of specific references and the interpretation of the literature is a possibility. We aimed to mitigate this with our extensive literature search and collective expertise regarding the clinical disease, genetic knowledge, and basic science experience. Thus, we present a comprehensive review of *CYP1B1*-related pediatric diseases and how our current understanding of the role of this gene based on animal studies may yield novel molecularly targeted treatments.

## Abbreviations

ARS	Axenfeld–Rieger syndrome
CCO	Congenital corneal opacity
CCS	Conserved core structures
CEU	Congenital ectropion uvea
DEAB	N,N-diethylaminobenzaldehyde
EET	Epoxyeicosatrienoic
HETE	Hydroxyeicosatetraenoic
JOAG	Juvenile open-angle glaucoma
PCG	Primary congenital glaucoma
POAG	Primary open-angle glaucoma
RA	Retinoic acid
VUS	Variant of uncertain significance
